# Supplementary material for: The Upstream 1350~1250 Nucleotide Sequences of the Human ENDOU-1 Gene Contain Critical Cis-Elements Responsible for Upregulating Its Transcription during ER Stress
Source: Int J Mol Sci. 2023 Dec 12;24(24):17393. doi: 10.3390/ijms242417393 (PMC10744159; doi:10.3390/ijms242417393)
Supplement: Supplementary file 1 [file ijms-24-17393-s001.zip › supplementary files/Supplementary files.pdf]

Table S1: TFsitescan transcription factor binding sites prediction results

	Site (Length)	PositionScore (Gaps)	Occurrence	EA Probability
EKLF_CS'(9)	26	7 (0)	0	2.87e-01
c-Myb-pseudorabies				
SREBP-1_CS'(9)	54	7 (0)	0	1.44e-01
C/EBP-beta_CS'(9)	88	7 (0)	0	2.87e-01
PAUSE-1_CS2(15)	137	10 (0)	0	1.00e00
CNTF_response_eleme				
JCV-B-1'(11)	159	10 (0)	0	1.00e00
MAZ-PTHR(9)	163	9 (0)	0	4.49e-03
MAZ_CS2(8)	164	7 (0)	0	7.18e-02
Sp1-a1p-inhibitor_(5'(11)	164	10 (0)	0	1.00e00
MAZ_CS2'(8)	168	7 (0)	0	7.18e-02
MAZ_CS2(8)	168	7 (0)	0	7.18e-02
CTF/NF1-CF1-2(11)	183	10 (0)	0	1.00e00
Yi-consensus'(10)	188	7 (0)	0	2.87e-01
apoE-PET'(12)	188	12 (0)	0	7.02e-05
APOE-PET(10)	190	10 (0)	0	1.12e-03
apoE-B1-II'(9)	190	9 (0)	0	4.49e-03
ApoE_B1'(9)	190	8 (0)	0	8.98e-03
APRT-human_US'(8)	192	8 (0)	0	1.80e-02
APRT-CHO_US'(8)	192	8 (0)	0	1.80e-02
ZFX-HLA-A11-1'(9)	194	9 (0)	0	4.49e-03
Yi-consensus'(10)	215	7 (0)	0	2.87e-01
AP-2_beta_CS'(9)	216	6 (0)	0	5.75e-01
AP1-ET-I(8)	228	8 (0)	0	1.80e-02
AP-1_CS1(8)	228	7 (0)	0	7.18e-02
claudin-CdxB'(11)	285	10 (0)	0	1.00e00
Rev-erb-alpha-CS(12)	291	10 (0)	0	1.12e-03
ROR-alpha-2_CS(12)	291	10 (0)	0	1.00e00
Rev-ErbAalpha_CS(11)	292	9 (0)	0	1.00e00
E2_RS1'(12)	316	8 (0)	0	2.87e-01
E2_RS1(12)	316	8 (0)	0	2.87e-01
BPV-E2_CS2'(12)	316	8 (0)	0	2.87e-01
BPV-E2_CS2(12)	316	8 (0)	0	2.87e-01
TFII-I-fos-SRE(11)	347	10 (0)	0	1.00e00
ANF-SRE/406'(12)	348	11 (0)	0	2.94e-01
PAI-2-TRM'(11)	356	10 (0)	0	1.00e00

AP-2_beta_CS(9)	363	6 (0)	0	5.75e-01
ssT1_CS(12)	376	8 (0)	0	1.44e-01
proximal_region/_A(8)	394	8 (0)	0	1.80e-02
AP1-TGF-beta1.2'(8)	412	8 (0)	0	1.80e-02
TEF1-GTI*'(9)	425	9 (0)	0	4.49e-03
Six5_CS(9)	458	7 (0)	0	7.18e-02
CP2-consensus(11)	530	8 (0)	0	7.18e-02
PuF_site(8)	540	8 (0)	0	1.80e-02
IgHC.8'(8)	630	8 (0)	0	1.80e-02
CuE3.1'(8)	630	8 (0)	0	1.80e-02
GBP/GAS-pIRE'(11)	654	10 (0)	0	1.00e00
Oct-X_CS(9)	669	7 (0)	0	7.18e-02
OTF-2A_RS(9)	669	9 (0)	0	4.49e-03
OTF-1_RS(9)	669	9 (0)	0	4.49e-03
Ig-dc.2'(10)	670	9 (0)	0	4.49e-03
Ig-kappa.2'(8)	670	8 (0)	0	1.80e-02
dc_element_CS'(10)	670	9 (0)	0	4.49e-03
cd_element_CS(10)	670	9 (0)	0	4.49e-03
Octa-U2snRNA(8)	670	8 (0)	0	1.80e-02
NF-A2-IgkLc'(8)	670	8 (0)	0	1.80e-02
NF-A1-IgkLc'(8)	670	8 (0)	0	1.80e-02
IgNF-A-Igk'(8)	670	8 (0)	0	1.80e-02
IgHC.16'(8)	670	8 (0)	0	1.80e-02
IgNF-A-IgH_(1)'(8)	670	8 (0)	0	1.80e-02
IgHC.15'(8)	670	8 (0)	0	1.80e-02
IgHC.14'(8)	670	8 (0)	0	1.80e-02
IgNF-A-IgH_(2)(8)	670	8 (0)	0	1.80e-02
IgHC.2'(8)	670	8 (0)	0	1.80e-02
NF-A2-IgHC'(8)	670	8 (0)	0	1.80e-02
NF-A1-IgHC'(8)	670	8 (0)	0	1.80e-02
IgHC.1'(8)	670	8 (0)	0	1.80e-02
histone-H2B-US'(8)	670	8 (0)	0	1.80e-02
dc_box.2'(10)	670	9 (0)	0	4.49e-03
OCTA3(8)	670	8 (0)	0	1.80e-02
OCTA1.1'(8)	670	8 (0)	0	1.80e-02
Ig_cd(10)	670	9 (0)	0	4.49e-03
dc_box'(10)	670	9 (0)	0	2.24e-03
HIF-1-guanylin'(11)	683	10 (0)	0	1.00e00

NF-E2-consensus_(1)'(8)	686	8 (0)	0	1.80e-02
AP1-TRE-4/C(9)	686	9 (0)	0	4.49e-03
NF-E2_CS2(9)	687	8 (0)	0	1.80e-02
AP-1_CS2(8)	687	8 (0)	0	1.80e-02
AP-2_beta_CS'(9)	694	6 (0)	0	5.75e-01
IL2-TCEp(10)	700	10 (0)	0	1.12e-03
Six5_CS(9)	714	7 (0)	0	7.18e-02
Sp1-GPC_(2)'(11)	731	10 (0)	0	1.00e00
Sp1-KDR/flk-1-IV(11)	732	10 (0)	0	1.00e00
Sp1-alpha-actin_(4)'(11)	732	10 (0)	0	1.00e00
KLF15-CS'(9)	733	7 (0)	0	7.18e-02
EKLF_CS'(9)	733	7 (0)	0	2.87e-01
KKLF_CS(9)	733	7 (0)	0	7.18e-02
MAZ_CS2'(8)	734	7 (0)	0	7.18e-02
MAZ_CS2(8)	734	7 (0)	0	7.18e-02
AP-2_beta_CS'(9)	762	6 (0)	0	5.75e-01
AP-2_beta_CS(9)	762	6 (0)	0	5.75e-01
AP-2_site'(9)	762	7 (0)	0	2.87e-01
AP-2_site(9)	762	7 (0)	0	2.87e-01
Olf-1-consensus(11)	781	10 (0)	0	2.24e-03
AP-2-p62_(2)(8)	790	8 (0)	0	1.80e-02
Ad5_EIA_element_I'(11)	799	10 (0)	0	1.00e00
E1A_element_I.2'(11)	799	10 (0)	0	1.00e00
MyoD-PRR-M-CAT(8)	822	8 (0)	0	1.80e-02
KLF15-CS(9)	841	7 (0)	0	7.18e-02
MAZ_CS2'(8)	841	7 (0)	0	7.18e-02
KKLF_CS'(9)	841	7 (0)	0	7.18e-02
IRF-1/pIRE'(11)	911	10 (0)	0	1.00e00
TTF-TPO-2'(8)	936	8 (0)	0	1.80e-02
T3R_TRE1_CS(8)	1064	7 (0)	0	7.18e-02
EKLF_CS'(9)	1072	7 (0)	0	2.87e-01
hIGFBP-1-HRE-2'(8)	1081	8 (0)	0	1.80e-02
CP1-gamma-globin_(3)(8)	1092	8 (0)	0	1.80e-02
PEA-3-TBXAS1(8)	1119	8 (0)	0	1.80e-02
MDR1-HT-p53-site-1'(11)	1209	10 (0)	0	1.00e00
MyoD_CS2'(9)	1233	7 (0)	0	1.44e-01
YY1_CS'(9)	1250	7 (0)	0	1.44e-01
ESE-3_CS(9)	1286	8 (0)	0	1.80e-02

Ets1-Fgf8(8)	1312	8 (0)	0	1.80e-02
c-Ets-2_CS'(8)	1312	7 (0)	0	7.18e-02
AP-2_beta_CS'(9)	1341	6 (0)	0	5.75e-01
AP-2_beta_CS(9)	1341	6 (0)	0	5.75e-01
AP-1_CS1(8)	1341	7 (0)	0	2.87e-01
ApoB-site_g(8)	1358	8 (0)	0	1.80e-02
PEA3_site_CS(8)	1360	7 (0)	0	3.59e-02
TCF-2-alpha_CS(8)	1360	7 (0)	0	7.18e-02
GABP_CS(8)	1361	7 (0)	0	3.59e-02
Ad5_EIA_element_I(11)	1361	10 (0)	0	1.00e00
E1A_element_I.2(11)	1361	10 (0)	0	1.00e00
AP2-FGFR4'(11)	1417	10 (0)	0	6.23e-01
AluA'(13)	1483	13 (0)	0	1.75e-05
delta-rpL7'(8)	1488	8 (0)	0	1.80e-02
AT-Alu(16)	1501	14 (0)	0	1.22e-03
Gfi-1-P21'(27)	1510	26 (0)	0	1.45e-10
HiNF-A_RS(12)	1538	9 (0)	0	1.80e-02
YY1_CS'(9)	1558	7 (0)	0	1.44e-01
YY1_CS4(11)	1559	9 (0)	0	1.00e00
PAI-2-CRE-element(8)	1560	8 (0)	0	1.80e-02
TSE/TRE/DR4_(1)'(16)	1560	12 (0)	0	1.56e-01
ESE-3_CS(9)	1587	8 (0)	0	1.80e-02
Ets1-Fgf8(8)	1587	8 (0)	0	1.80e-02
c-Ets-2_CS'(8)	1587	7 (0)	0	7.18e-02
SREBP-1_CS(9)	1594	7 (0)	0	1.44e-01
AP-2-p62_(1)'(8)	1614	8 (0)	0	1.80e-02
Gfi-1-AZU-1(30)	1617	29 (0)	0	2.27e-12
Gfi-1-ACT'(30)	1618	28 (0)	0	2.51e-09
LUN_RS'(16)	1618	16 (0)	0	2.74e-07
PLOD1-Bic-NKE'(15)	1622	14 (0)	0	2.43e-03
HIOMT-A-E4(18)	1625	17 (0)	0	3.80e-05
bicoid-CAMLG(8)	1631	8 (0)	0	1.80e-02
C/EBP-TTRS3'(8)	1643	8 (0)	0	1.80e-02
C/EBP_CS2'(8)	1643	7 (0)	0	7.18e-02
TTF-1-SP-D(11)	1642	10 (0)	0	6.23e-01
E47-MyoD_CS'(8)	1646	7 (0)	0	5.39e-02
MT-IG-MRE-b'(13)	1651	12 (0)	0	3.90e-02
GABP-Slp_site'(8)	1659	8 (0)	0	1.80e-02

Elk-1_CS(8)	1661	7 (0)	0	7.18e-02
IL2R-Ets(8)	1661	8 (0)	0	1.80e-02
Initiator_CS'(8)	1677	7 (0)	0	7.18e-02
Initiator_CS'(8)	1717	7 (0)	0	7.18e-02
crystallin-DE1A'(11)	1728	10 (0)	0	6.23e-01
dc_box.2(10)	1745	9 (0)	0	4.49e-03
Ig_cd'(10)	1745	9 (0)	0	4.49e-03
dc_box(10)	1745	9 (0)	0	4.49e-03
Ig-dc.2(10)	1745	9 (0)	0	2.24e-03
dc_element_CS(10)	1745	9 (0)	0	4.49e-03
cd_element_CS'(10)	1745	9 (0)	0	4.49e-03
Oct-X_CS'(9)	1747	7 (0)	0	4.49e-03
OTF-2A_RS'(9)	1747	9 (0)	0	4.49e-03
OTF-1_RS'(9)	1747	9 (0)	0	4.49e-03
Ig-kappa.2(8)	1747	8 (0)	0	1.80e-02
Octa-U2snRNA'(8)	1747	8 (0)	0	1.80e-02
NF-A2-IgkLc(8)	1747	8 (0)	0	1.80e-02
NF-A1-IgkLc(8)	1747	8 (0)	0	1.80e-02
IgNF-A-Igk(8)	1747	8 (0)	0	1.80e-02
IgHC.16(8)	1747	8 (0)	0	1.80e-02
IgHC.15(8)	1747	8 (0)	0	1.80e-02
IgHC.14(8)	1747	8 (0)	0	1.80e-02
IgNF-A-IgH_(2)'(8)	1747	8 (0)	0	1.80e-02
IgHC.2(8)	1747	8 (0)	0	1.80e-02
NF-A2-IgHC(8)	1747	8 (0)	0	1.80e-02
NF-A1-IgHC(8)	1747	8 (0)	0	1.80e-02
IgHC.1(8)	1747	8 (0)	0	1.80e-02
IgNF-A-IgH_(1)(8)	1747	8 (0)	0	1.80e-02
histone-H2B-US(8)	1747	8 (0)	0	1.80e-02
OCTA3'(8)	1747	8 (0)	0	1.80e-02
OCTA1.1(8)	1747	8 (0)	0	1.80e-02
IRF-2_RS'(8)	1758	6 (0)	0	2.87e-01
STE6.2(8)	1762	8 (0)	0	1.80e-02
CK-8-mer(8)	1770	7 (0)	0	7.18e-02
RIPE3b'(8)	1782	8 (0)	0	1.80e-02
Ets1-erk1'(8)	1812	8 (0)	0	1.80e-02
TCF-1alpha-ADA(11)	1866	10 (0)	0	6.23e-01
AP-2-cyclinD2_(1)(8)	1866	8 (0)	0	1.80e-02

AP-2-erk1(9)	1889	9 (0)	0	4.49e-03
C/EBP-beta_CS(9)	1917	7 (0)	0	2.87e-01
Math3-E-box(8)	1942	8 (0)	0	1.80e-02
CP2-consensus'(11)	1965	8 (0)	0	7.18e-02
CK-8-mer(8)	1972	7 (0)	0	7.18e-02
keratinocyte_CS(8)	1972	7 (0)	0	3.59e-02
MEIS1_CS'(8)	1979	7 (0)	0	1.44e-01
Oct-1-IE-3_(3)(9)	2075	9 (0)	0	4.49e-03
AP-2-TBXAS1(8)	2110	8 (0)	0	1.80e-02

Table S2. List of oligo primers used in this study

Primer name	Sequence (5'→3')	Used in plasmid construction
hE-1p-2.1F	TTCTCTATCGATAACGCGTTTGCTCCTGACCTAAAATTCCATTAATGGG	pE2.1p
hE-1p-2.1R	CGAGCCCGGGCTAGCAAGCTTGGTGCCCAGTTGGAGGCCAAAAAGG	pE2.1p
F1	GCAGTGAGCCGAGATTGCCCTAGGCTTGTCCTTCT	pE2.1p-d1350/1250
R1	ATCTCGAGTTTTCCTGCCCATGCGCCAGGT	pE2.1p-d1350/1250
F2	AAGGTACCGGAGTCATCCTTAACTGTCTTCCCCAGC	pE2.1p-d1350/1250
R2	GACAAGCCTAGGGCAATCTCGGCTCACTGCAACCT	pE2.1p-d1350/1250
F3	AAGGTACCGGAGTCATCCTTAACTGTC	pE2.1p-d1485/1350
R3	GGCTGGATGCAGCAGTGTGACCTGATCTCAAACTCTTGA	pE2.1p-d1485/1350
F4	TCAAGAGTTTGAGATCAGGTCACACTGCTGCACTCCAGCC	pE2.1p-d1485/1350
R4	AAAGGCCTCTTAAAGGCAGCCGAAGGC	pE2.1p-d1485/1350
F5	AAGGTACCGGAGTCATCCTTAACTGTC	pE2.1p-d1649/1486
R5	CCAATGGCTGCTTCATTTGCAT	pE2.1p-d1649/1486
F6	TCAGAGACACGTTTATTCTGCTCTTACTCA	pE2.1p-d1749/1650
R6	ATCTCGAGTTTTCCTGCCCATGC	pE2.1p-d1749/1650
F7	AAGGTACCGGAGTCATCCTTAACTGTCTTCC	pE2.1p-d1749/1650
R7	TGAGTAAGAGCAGAATAAACGTGTCTCTGACTCCT	pE2.1p-d1749/1650
F8	GACACTTGGAAGGGATGTGGTGCCAACCCAGGGAG	pE2.1p-d1850/1750
R8	ATCTCGAGTTTTCCTGCCCATGCGCCAGGTTGAACAGTTGCTGGTGGGTTA	pE2.1p-d1850/1750
F9	GGAACCGGAGTCATCCTTAACTGTCTTCC	pE2.1p-d1850/1750
R9	TGGGTTGGCACCACATCCCTTCCAAGTGTCCAGCT	pE2.1p-d1850/1750

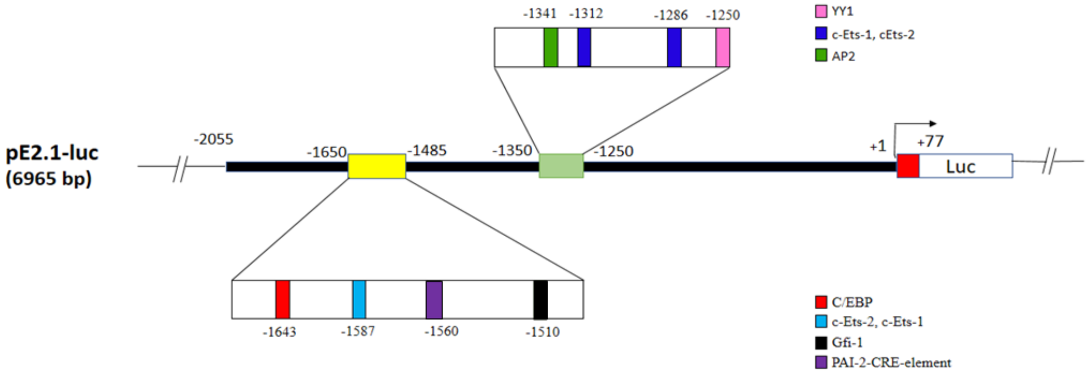

Figure S1. TFBSs within the -1650~-1485 and -1350~-1250 segments of human *ENDOU-1* gene, as predicted using TFsitescan.

A

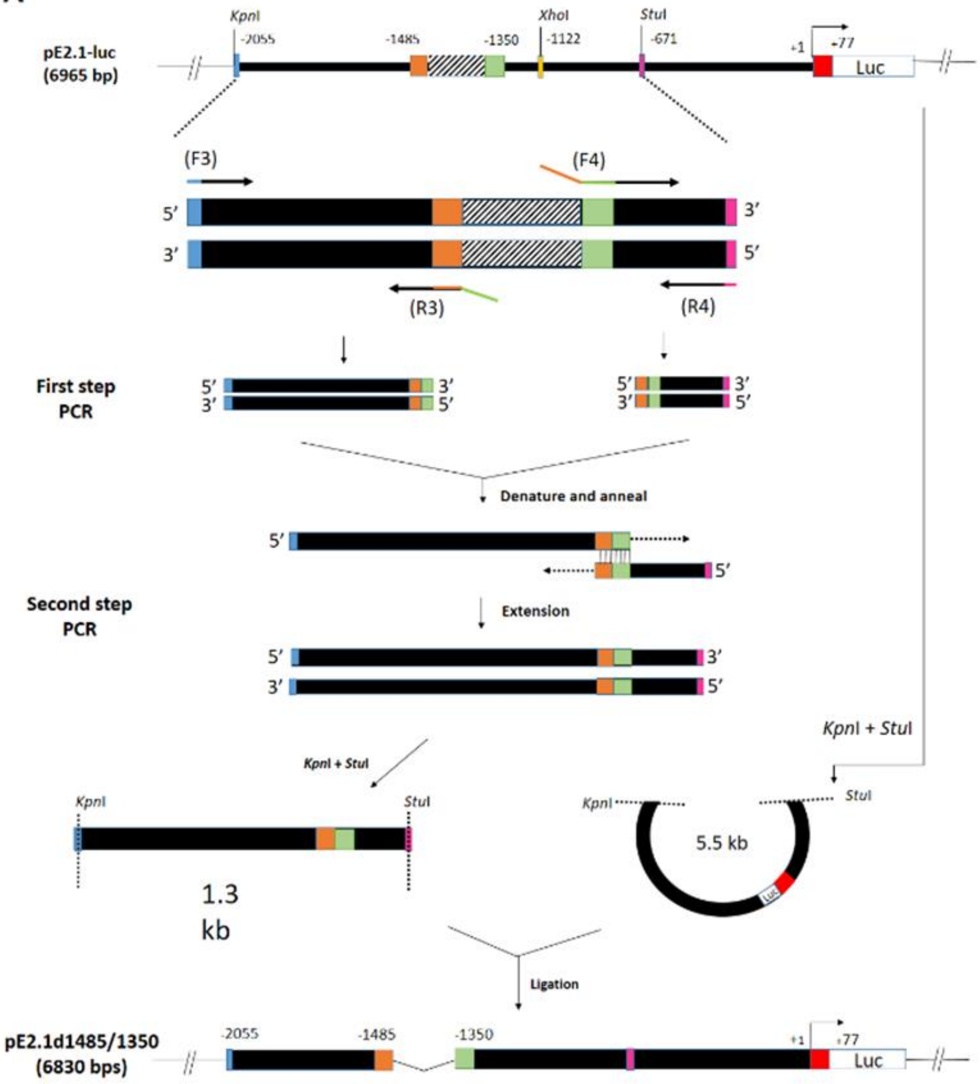
pE2.1-luc
(6965 bp)
KpnI
-2055
-1485
-1350
XhoI
-1122
StuI
-671
+1
+77
Luc
(F3)
(F4)
5′
3′
3′
5′
(R3)
(R4)
First step
PCR
Denature and anneal
Second step
PCR
Extension
KpnI + StuI
KpnI + StuI
KpnI
StuI
1.3
kb
5.5 kb
Luc
Ligation
pE2.1d1485/1350
(6830 bps)
-2055
-1485
-1350
+1
+77
Luc

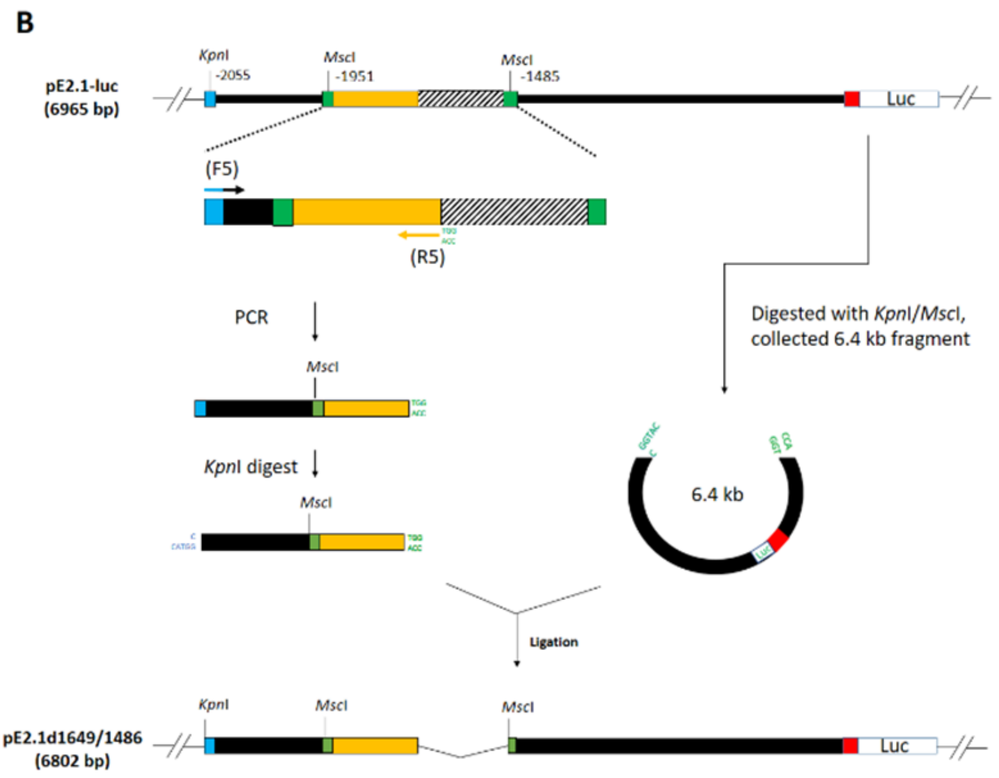

Figure S2. Scheme of plasmid construction. (A) Two-step PCR strategy to generate plasmids harboring various internal deletions within the pE2.1p construct. For example, pE2.1p lacking -1485~-1350 (pE2.1p-d1485/1350). Plasmids pE2.1p-d1850/1750 and pE2.1p-d1749/1650 were generated using the same strategy except of using restriction enzymes KpnI and XhoI and corresponding primers (Table 1). Similarly, pE2.1p-d1350/1250 was generated using XhoI and StuI and corresponding primers. (B) Restriction and ligation strategy to generate plasmid pE2.1p-d1649/1486. A 0.5 kb PCR product containing an internal MscI cutting site was amplified using primers F5 and R5, followed by insertion into KpnI and MscI-digested plasmid pE2.1p.
