# Supplementary figures and images for: The Upstream 1350~1250 Nucleotide Sequences of the Human ENDOU-1 Gene Contain Critical Cis-Elements Responsible for Upregulating Its Transcription during ER Stress
Source: Int J Mol Sci. 2023 Dec 12;24(24):17393. doi: 10.3390/ijms242417393 (PMC10744159; doi:10.3390/ijms242417393)

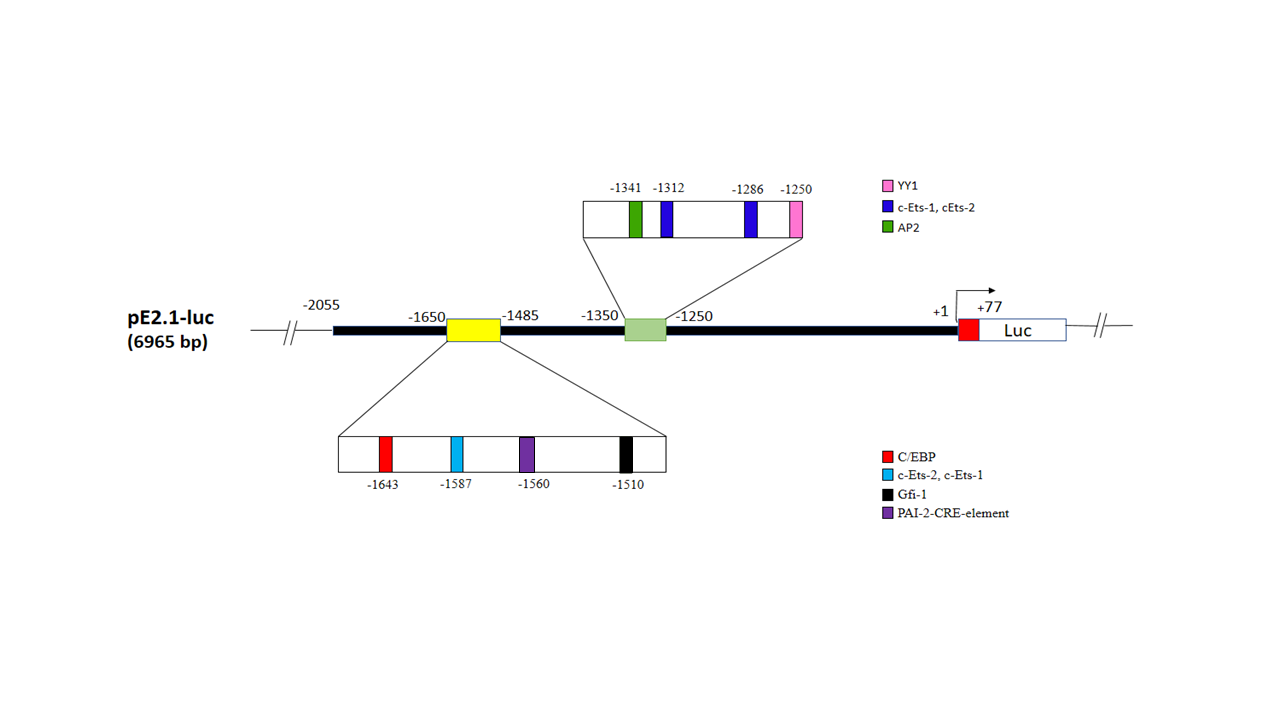

Supplement: Supplementary file 1 [file ijms-24-17393-s001.zip › supplementary files/Figure S1.tif]

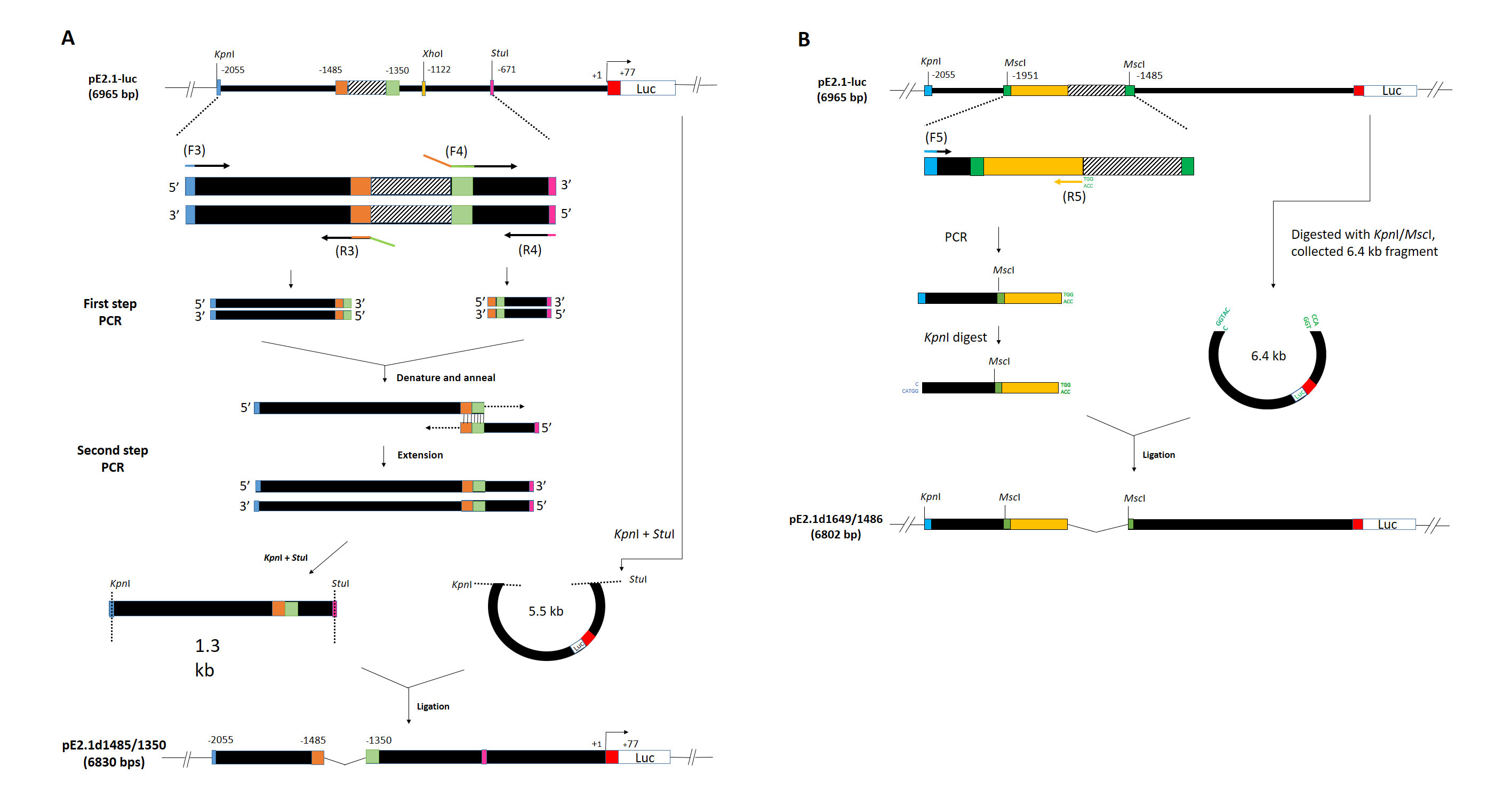

Supplement: Supplementary file 1 [file ijms-24-17393-s001.zip › supplementary files/Figure S2.tif]
